# Reversal of freshening trend of Antarctic Bottom Water in the Australian-Antarctic Basin during 2010s

**DOI:** 10.1038/s41598-020-71290-6

**Published:** 2020-09-15

**Authors:** S. Aoki, K. Yamazaki, D. Hirano, K. Katsumata, K. Shimada, Y. Kitade, H. Sasaki, H. Murase

**Affiliations:** 1 Institute of Low Temperature Science, Hokkaido University, Sapporo, Japan; 2 Graduate School of Environmental Science, Hokkaido University, Sapporo, Japan; 3 Arctic Research Center, Hokkaido University, Sapporo, Japan; 4 Japan Agency for Marine-Earth Science and Technology, Yokosuka, Japan; 5 Tokyo University of Marine Science and Technology, Tokyo, Japan; 6 Japan Fisheries Research and Education Agency, Yokohama, Japan

**Keywords:** Ocean sciences, Climate sciences, Climate change, Ocean sciences

## Abstract

The Antarctic continental margin supplies the densest bottom water to the global abyss. From the late twentieth century, an acceleration in the long-term freshening of Antarctic Bottom Waters (AABW) has been detected in the Australian-Antarctic Basin. Our latest hydrographic observations reveal that, in the late 2010s, the freshening trend has reversed broadly over the continental slope. Near-bottom salinities in 2018–2019 were higher than during 2011–2015. Along 170° E, the salinity increase between 2011 and 2018 was greater than that observed in the west. The layer thickness of the densest AABW increased during the 2010s, suggesting that the Ross Sea Bottom Water intensification was a major source of the salinity increase. Freshwater content on the continental slope decreased at a rate of 58 ± 37 Gt/a in the near-bottom layer. The decadal change is very likely due to changes in Ross Sea shelf water attributable to a decrease in meltwater from West Antarctic ice shelves for the corresponding period.

## Introduction

Cold and dense water on the continental shelf around Antarctica feeds the abyssal waters of the global oceans to produce Antarctic Bottom Water (AABW)^1^, which plays a crucial role in global mass, heat and freshwater transport^2^. Prominent and long-term changes in the properties of AABW have been found in the Southern Ocean from the late twentieth century^3^, and freshening and warming in the Southern Ocean is known to be widespread^4–6^. Changes in the AABW properties are potentially related to the changes in global overturning circulation and directly affect the abyssal contribution to sea level rise.

Among the major abyssal basins around Antarctica, the Australia-Antarctic Basin (AAB) off East Antarctica is located to the west of the Southeast Pacific Basin, encompassing West Antarctica. AABW in the Ross Sea (Ross Sea Bottom Water; RSBW), formed by intense sea-ice production on the continental shelf followed by dense shelf water outflow and mixing with ambient deep water, flows westward into the AAB^7^. Off the Adélie/George V Land (AGVL) Coast, AABW is formed through the export of dense shelf water, originating from intense sea-ice production in the George V Land polynya system, which eventually joins the bottom water originating in the Ross Sea^7^. AABW flows equator-ward as the deep western boundary current^8^, partly extending into the Princess Elizabeth Trough (PET).

From the latter half of the twentieth century to the early 2010s, changes have been observed in the water mass properties of AABW in the AAB. An accelerated freshening of AABW in the period from 1995 to 2005, when compared to the period 1960/70s–1995, is reported^9^. The volume of AABW decreased from the 1970s to 2008–2012, with the largest signal found in the east^10^. The freshening of AABW continued to early 2010s in the AAB^11^. Regional and interannual variations in AABW are also evident. Along 140° E, the Adélie Land Bottom Water freshened until 2012, when its layer thickness was anomalously thin, soon after the calving event of the Mertz Glacier Tongue^12^. From the 2000s to mid-2010s, in the western end of the AAB, the freshening of AABW further accelerated^13^. Moreover, freshening of RSBW, which was detected from the latter half of the twentieth century until the 2000s, mostly affects the freshening of AABW in the AAB^14–16^. However, the accelerated freshening may not last long, because it depends on unknown variability in several potential sources. In the high-latitude North Atlantic Ocean, which is another source region of deep water, decadal and multi-decadal variability are vigorous in comparison, and advection plays a role in carrying the variability^3^.

Dense shelf water, which is one of the source waters of AABW, is sensitive to the various air-sea-ice fluxes, and hence the temporal changes in the characteristics of dense shelf water can lead to the changes in AABW^15^. The freshening of the shelf water in the Ross Sea until the late 2000s is further attributed to the change in melt water flux upstream in West Antarctica^17^. The calving event of the Mertz Glacier Tongue in the Adélie Depression changed the sea-ice production and subsequently the salinity of the shelf water of the Adélie Depression^18^. Recently, the presence of decadal variability in the melt water and continental ice fluxes was revealed in the Amundsen Sea^19,20^. Salinity on the continental shelf of the Ross Sea increased from the early 2010s after the long-term decrease from the 1950s^21^. The decadal variabilities on the shelf can significantly affect the property changes of AABW^22^. However, the presence of decadal variability of AABW, especially up to date, is not known. Hence, temporally high-resolution hydrographic observations over a broad area are important to describe and predict the tendency and spread of the salinity change signal.

**Figure 1:**
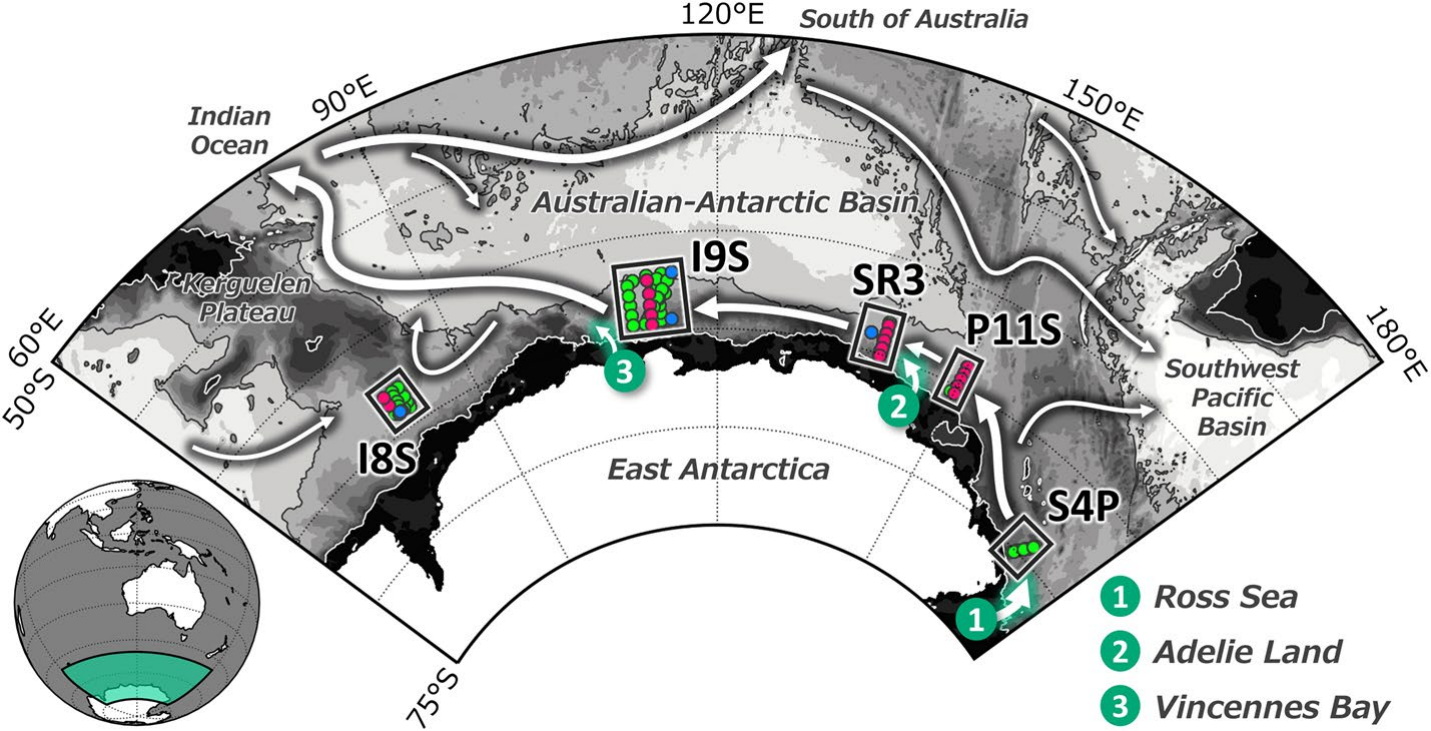
Schematic of the Antarctic Bottom Water ventilation (green arrows) and pathway (black arrows) in the Australian-Antarctic Basin, based on the work of Orsi et al.^1^, Rintoul^7^, Heywood et al.^31^, Kitade et al.^32^, McCartney and Donohue^33^, and van Wijk and Rintoul^10^. The observation stations used in this study are indicated by dots. Red, blue and green dots denote observational locations by the R/V Kaiyo-maru, R/V Eltanin, and other vessels, respectively. White (black) line denotes 1,000 m (4,000 m) isobath.

Based on the large-scale hydrographic array obtained by R/V Kaiyo-maru in 2018/19, which was designed to revisit the array occupied in 1996^23^, and other high-quality hydrographic sections in the AAB, we constructed a pentadal time series from the 1990s to reveal the spatio-temporal variability of the AABW salinity on the continental slope of five sections between 170° E and 80° E (Fig. 1). The new observational array in the latter half of the 2010s reveals a change in salinity trend in this region.

## Results

**Figure 2:**
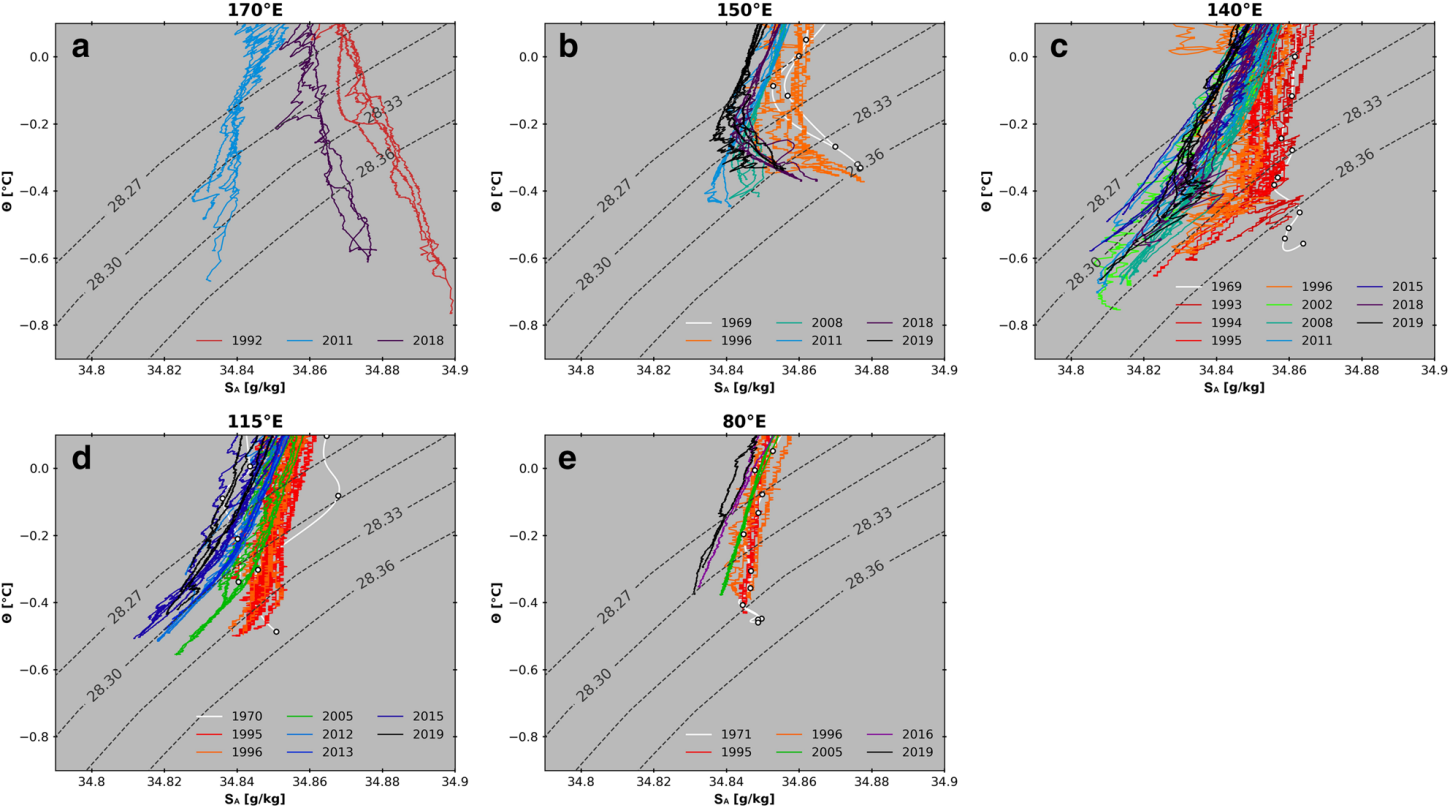
Temporal evolution of Antarctic Bottom Water properties in the Australian-Antarctic Basin. Conservative temperature (Ɵ) and Absolute Salinity (S_A_) plots are shown in order of the AABW pathway: (**a**) 170° E; (**b**) 150° E; (**c**) 140° E; (**d**) 115° E; (**e**) 80° E.

Significant freshening occurred in benthic water masses over a wide area in the AAB until the early 2010s, but the latest observations reveal that this tendency has reversed (Fig. 2). At 150° E, a strong salinity maximum present near the bottom in 1969/1996, which indicates the contribution of RSBW, was almost eroded in 2008/2011 (Fig. 2b). However, in 2018 and 2019, the benthic salinity maximum was again evident. Hence, the reversal of the benthic signal occurred sometime between 2011 and 2018. At 140° E, a slightly saline feature was detected near the bottom in 1969 but the near-bottom salinity decreased in the 1990s (Fig. 2c). Freshening events were evident in the 2000s and 2011–2015. However, the near-bottom salinity became slightly more saline in 2018 and 2019. At 115° E, freshening was evident near the bottom from the 1990s to early 2010s, and the recorded bottom salinity was minimal in 2015 (Fig. 2d). Then, near-bottom salinity increased from 2015 to 2019.

Although the temporal resolution is sparse, salinity changed significantly in the upstream Ross Sea at 170° E (Fig. 2a). The salinification towards the bottom in 1992 decreased in 2011, but the saline feature was evident again in 2018, suggesting the presence of significant decadal variability. On the other hand, downstream in the northern PET (80° E), changes near the bottom are less obvious (Fig. 2e). Freshening continued from the 1970s to 2019 with a range much smaller than those in the easterly sections.

**Figure 3:**
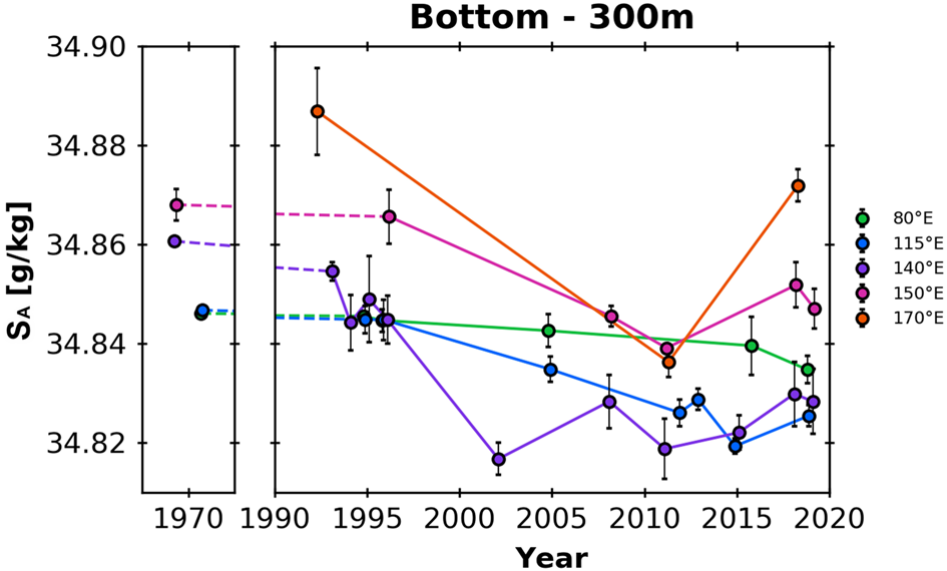
Changes in the Absolute Salinity averaged over the bottom 300 dbar at 170° E (orange), 150° E (pink), 140° E (purple), 115° E (blue), and 80° E (green). The error bars denote the standard deviation among the points used.

To quantify the salinity changes near the bottom, a vertical average over the bottom 300 dbar, which is a typical length scale associated with a downslope flow^22,24^, was examined (Fig. 3). The minimum in salinity occurred sometime in the early to the middle 2010s at most sections along the continental slope of the AAB. At 170° E, salinity was minimal in 2011 and increased by 0.036 ± 0.003 (the standard error of the difference between means) by 2018. At 150° E, salinity was again minimal in 2011 and increased by 0.013 ± 0.003 in 2018 and 0.008 ± 0.003 in 2019. At 140° E, salinity was at a low level in 2011 and 2015, although a minimum was recorded in 2002. From 2015, it increased by 0.008 ± 0.005 in 2018 and by 0.006 ± 0.005 in 2019. At 115° E, 2015 was the year of salinity minimum. Salinity then increased in 2019 by 0.006 ± 0.002. At 80° E, moderate freshening continued, which was different from the other eastern sections. Salinity was fresher in 2019 than in 2005, although there was no record available in the first pentad of the 2010s. Overall, a minimum in salinity was recorded in the early 2010s, increasing towards the late 2010s. The rate of increase in the 2010s are higher to the east.

**Figure 4:**
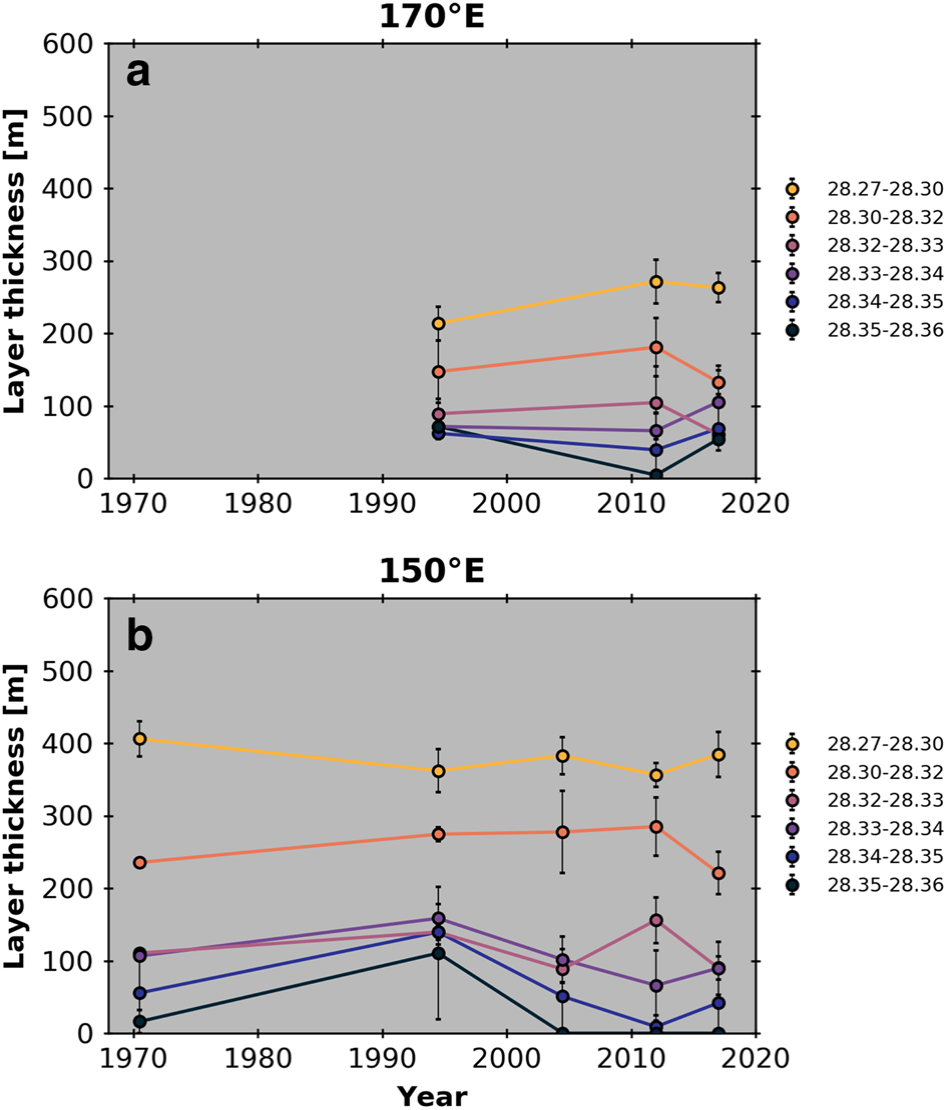
Changes in the average thickness of individual density layers for AABW (γ^n^ ≥ 28.27 kg m^−3^) at (**a**) 170° E and (**b**) 150° E. The error bars denote the standard deviation among the points used.

The freshening of AABW was accompanied by a volume contraction of dense AABW layers^25^. The contraction trend of the densest AABW layer also changed in the late 2010s (Fig. 4). For 170° E and 150° E, the layer thickness of the densest waters at 28.33–28.36 kg m^−3^ increased from 2015 to 2018/19 after the decrease from the 1990s to early 2010s. The increase in layer thickness indicates the increase in volume of RSBW during the latter half of the 2010s.

**Figure 5:**
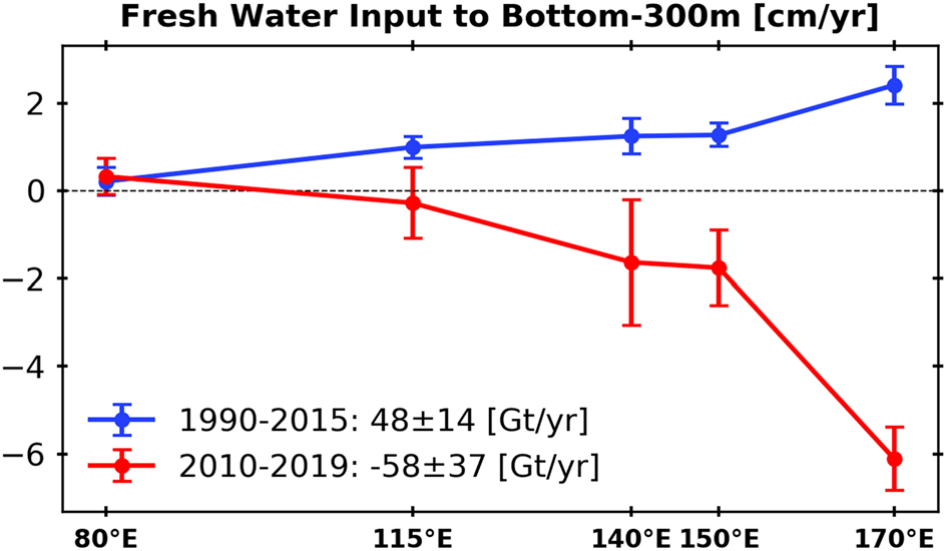
Freshwater content change (cm a^−1^) over the bottom 300 dbar in the Australian-Antarctic Basin from 170° E to 80° E during 1990–2015 (blue) and during 2010–2019 (red). The error bars denote the standard deviation of the trend (see Methods).

To quantify the reversal of the salinity tendency in the bottom 300 dbar layer, the salinity trends before and after the early 2010s were calculated at each section from the pentadal average and derived change in freshwater content ("Methods" section; Fig. 5). The freshening trend from 1990s to the early 2010s was larger upstream and gradually decreased to the west. A simple box calculation with a 200 km width and 300 dbar height was conducted for about 4,200 km distance from 170° E to 80° E along the continental slope of 2,500–4,000 m deep. During the period from 1995 to the early 2010s, freshwater content increased by about 48 ± 14 Gt/a, which is roughly consistent with a previous study^9^. As for the salinity reversal during the former 2010s to 2019, the general decrease in the amplitude of the signal is robust, and the freshwater content decreased by 58 ± 37 Gt/a. The absolute magnitude of decrease in freshwater content in the 2010s was comparable to that of the increase during 1990s–early 2010s.

## Discussion

Given the spatial distribution of the magnitude of the salinity reversal in the late 2010s, a major source of the signal is not in the west but in the east of the AAB, as in the case of freshening until the 2010s. Although the temporal resolution is not enough to identify a specific year, it is reasonable to assume that the salinity minimum occurred sometime within the period 2011–2015 upstream in the east, to be consistent with the overall AABW change downstream in the AAB.

Assuming that the salinity change of the RSBW at 170° E (about 0.005 a^−1^ increase over seven years during 2011–2018) reflects the change in shelf water property, the increase rate of the shelf water salinity in the Ross Sea in the 2010s is considered to be about 0.01 a^−1^ during the corresponding period (with mixing ratio of dense shelf water : mCDW = 1:2), which is roughly consistent with the estimates of 0.015–0.0025 a^−1^ observed at Drygalski Trough mouth, Joides Trough, and Terra Nova Bay during 2014–2018^21^. The estimated magnitude is significantly larger than the long-term decrease of 0.003 a^−1^ in shelf water salinity during 1958–2008^15,21^. There are multiple factors that can cause a change in shelf water property; such as changes in local sea-ice production, cross-slope exchange, and advection. Recently, a reduction of meltwater flux was revealed upstream in West Antarctica; at the ice front of the Dotson Ice Shelf, the melt flux was about 80 Gt/a in 2009 and then it decreased to about 20 Gt/a in 2012^20^. A similar change is possible for the nearby ice shelves. Since the melt water takes a few years to flow westward into the Ross Sea and contribute to bottom water formation, the magnitude and the timing is consistent with the salinity increase in AABW in the AAB. On the other hand, the increase in meltwater flux from West Antarctica in the 2000s, before the reduction, can also be consistent with the freshening acceleration during 2007–2016 when compared to 1995–2007 off the Kerguelen Plateau^13^.

The effect of freshwater export from the AGVL Coast on the AABW properties is complex and not robustly identified. Salinity was very low in 2002 (Fig. 3). Salinity of the dense shelf water decreased associated with the reduction in sea-ice production after the calving event of the Mertz Glacier Tongue in 2010^18,26,27^. After 2011, salinity increases in some regions were observed, possibly due to polynya formation at the lee side of the relocated large iceberg^28^. Although the interannual variability could be large in the AABW salinity at 140° E, the magnitude of decadal or longer-term changes in salinity and hence freshwater content was comparable to those at 150° E (Fig. 3). Hence, the effect of the upstream dominates, over the changes of AGVL Coast origin, the overall basin-scale change on the decadal time scale.

Our study demonstrates that the salinity of AABW in the AAB increased on the decadal time scale in the last decade. Given the presence of decadal variability on the continental shelf, this finding indicates that sampling with a time interval of more than 10 years is not enough to resolve and describe the development of AABW properties. The recent advent of float technology such as Deep Argo enables a higher-temporal sampling of bottom water^29^. The combination of sustained hydrographic and moored observations and Lagrangian observations are effective in fully describing the development of freshwater transport by AABW.

## Methods

### Data source

We used high-quality top-to-bottom hydrographic observations in the AAB from the 1990s until 2019. The continental slopes of five sections at 170° E, 150° E, 140° E, 115° E, and 80° E were studied (Supplementary Table S1). The R/V Kaiyo-maru, Fisheries Agency of Japan, conducted a research cruise over 80°–150° E in the AAB, in the proximity of its seasonal ice edge, from December 2018 to March 2019. Previously in 1996, this part of the AAB was observed simultaneously by the R/V Aurora Australis^21^. Data from the section 115° E is supplemented by data obtained by the Training and Research Vessel Umitaka-maru, Tokyo University of Marine Science and Technology, obtained in 2015. CTD data from the WOCE Hydrographic Program (WHP) repeat and nearby sections from the 1990s are also compiled. A total of 20 cruises in the austral summer from December to March were used. The year in the text denotes the new year of the season (i.e. the cruise that took place during December 2018–March 2019 is treated as 2019). The data were collected according to the WHP/GO-SHIP standards^30^.

Data were selected for the five sections on the continental slope with a depth range of 2,500–4,000 m. Since the deepest depth was around 3,000 m along the 170° E section, data were selected from the depth range of 2,500–3,000 m.

Although the overall sampling interval is not homogeneous, all sections (except 80° E and 170° E) covered CTD data in the 1990s, 2000s and former/latter pentads of the 2010s. Data along 170° E separate the former and latter half of the 2010s, although the section was visited only three times in total. Data along 80° E were not sampled in the early 2010s. In the 1970s, historical cruises by R/V Eltanin are supplemented for the four sections spanning from 80° E to 150° E.

### Data processing

We constructed time series for AABW properties for the five sections. The temporal sampling interval was not homogeneous among the sections as described above. To filter out the variability with time scales of a few years, station data were averaged within five periods (I. 1969–1971, II. 1990–1999, III. 2000–2009, IV. 2010–2015, and V. 2016–2019) in calculating the density layer thickness (Fig. 4) and freshwater content change (Fig. 5). The duration is represented by the central time in each period.

We estimated the freshwater flux per unit area *V*_*fw*_ by a standard method^5^. Initially, a water column on a unit area and a height *H*_1_ has a salinity *S*_1_. After adding a freshwater *V*_*fw*_ over a period *dt* at the surface and mixing, the column increased in height to become *H*_2_ and a salinity *S*_*2*_. Since the salt content is conserved,

$$ V_{fw} = (H_{2} - H_{1} ) / dt = \left( {\frac{{S_{1} }}{{S_{2} }} - 1} \right)H_{1} / dt, $$

where we neglected the small change in density. In Fig. 5, *V*_*fw*_ for 1990–2015 was calculated by the salinity trend between the second and fourth periods, and *V*_*fw*_ for 2010–2019 was calculated by the trend between the fourth and fifth periods (for 80° E, the lacking fourth period was substituted by the third period).

The estimate was derived for the layer thickness *H*_*1*_ (300 dbar) at the bottom. Using the mean and standard deviation of salinity estimate in each period, 1,000 random sets of salinity time series were artificially generated. The mean and standard deviation of V_fw_ was then estimated from the 1,000 datasets. An estimate of total freshwater flux over the domain was calculated by integrating V_fw_ in zonal direction and then multiplied by 200 km, which roughly corresponds to the meridional distance between the 2,500 and 4,000 m isobaths on the continental slope.

## Supplementary information

Supplementary file1

## Data Availability

Hydrographic data from Kaiyo-maru are available through CCHDO (https://cchdo.ucsd.edu/). Hydrographic data from WHP S4P, P11S, SR3, I9S, I8S, and BROKE are available through CCHDO (https://cchdo.ucsd.edu/). Hydrographic data from Umitaka-maru will be available through the NIPR ADS database (https://ads.nipr.ac.jp/).

## Code availability

The Python scripts used to analyse the data and to generate the figures are available from the corresponding author on request.
