# Supplementary material for: Reversal of freshening trend of Antarctic Bottom Water in the Australian-Antarctic Basin during 2010s
Source: Sci Rep. 2020 Sep 15;10:14415. doi: 10.1038/s41598-020-71290-6 (PMC7492216; doi:10.1038/s41598-020-71290-6)
Supplement: Supplementary file 1 — Supplementary file1 [file 41598_2020_71290_MOESM1_ESM.pdf]

Supplementary Information

Reversal of freshening trend of Antarctic Bottom Water in the Australian-Antarctic Basin during 2010s

S. Aoki*[1,2], K. Yamazaki[2], D. Hirano[1,2,3], K. Katsumata[4], K. Shimada[5], Y. Kitade[5], H. Sasaki[6], and H. Murase[5]

[1]Institute of Low Temperature Science, Hokkaido University, Sapporo, Japan.

[2]Graduate School of Environmental Science, Hokkaido University, Japan.

[3]Arctic Research Center, Hokkaido University, Japan.

[4]Japan Agency for Marine-Earth Science and Technology, Yokosuka, Japan.

[5]Tokyo University of Marine Science and Technology, Tokyo, Japan.

[6]Japan Fisheries Research and Education Agency, Yokohama, Japan.

*Corresponding author: shigeru@lowtem.hokudai.ac.jp

Supplementary
Table S1

Supplementary table S1. Summary of CTD observation used in the Australian-Antarctic Basin.

year	month	80°E	115°E	140°E	150°E	170°E	Platform	Expocode
1969	3			○	○		Eltanin	N/A
1971	2	○	○				Eltanin	N/A
1992	3					○	Akademik Ioffe	90KDIOFFE6_1
1993	3			○			Aurora Australis	09AR9309_1
1994	1			○			Aurora Australis	09AR9407_1
1995	1		○	○			Aurora Australis	09AR9404_1
1995	1	○	○				Knorr	316N145_5
1996	2	○	○	○	○		Aurora Australis	09AR9604_1
2001	12			○			Aurora Australis	09AR20011029
2005	1	○	○				Aurora Australis	09AR20041223
2008	1			○	○		Aurora Australis	09AR20071216
2008	3			○			Aurora Australis	09AR20080322
2011	1			○	○		Aurora Australis	09AR20110104
2011	2					○	Nathaniel B. Palmer	320620110219
2012	1		○				Aurora Australis	09AR1203_1
2012	12		○				Mirai	49NZ20121128
2015	1			○			Aurora Australis	N/A
2015	1		○				Umitaka-maru	N/A
2016	2	○					Roger Revelle	33RR20160208
2018	1			○	○		Investigator	096U20180111
2018	3					○	Nathaniel B.Palmer	3210620180309
2019	3	○	○	○	○		Kaiyo-maru	490S20181205
